# Surgery-related predictors of kneeling ability following total knee arthroplasty: a systematic review and meta-analysis

**DOI:** 10.1186/s43019-021-00117-z

**Published:** 2021-10-02

**Authors:** Shaheer Nadeem, Raman Mundi, Harman Chaudhry

**Affiliations:** 1 Faculty of Health Sciences, McMaster University, 1280 Main St W, Hamilton, ON L8S 4L8 Canada; 2 Division of Orthopaedic Surgery, University of Toronto, 149 College Street, Toronto, ON M5T 1P5 Canada; 3 Sunnybrook Holland Orthopaedic and Arthritic Centre, 43 Wellesley St E, Toronto, ON M4Y 1H1 Canada

**Keywords:** Arthroplasty, Kneel, Knee replacement, Primary

## Abstract

**Purpose:**

Kneeling ability is among the poorest outcomes following total knee arthroplasty (TKA). The purpose of this meta-analysis was to: (1) quantify kneeling ability after TKA; (2) identify surgical approaches and prosthesis designs that improve kneeling ability following TKA; and (3) quantify the effectiveness of these approaches.

**Methods:**

We performed a systematic review in accordance with the PRISMA guidelines of multiple medical databases. Data relating to demographics, TKA technique, prosthesis design, and kneeling-specific outcomes were extracted. Comparative outcomes data were pooled using a random effects model.

**Results:**

Thirty-six studies met the eligibility criteria. The proportion of patients able to kneel increased with longer follow-up (36.8% at a minimum of 1 year follow-up versus 47.6% after a minimum of 3 years follow-up, *p* < 0.001). The odds of kneeling were greater for patients undergoing an anterolateral incision compared with an anteromedial incision (OR 3.0, 95% CI 1.3–6.9, *p* = 0.02); a transverse incision compared with a longitudinal incision (OR 3.5, 95% CI 1.4–8.7, *p* = 0.008); and a shorter incision compared with a longer incision (OR 8.5, 95% CI 2.3–30.9, *p* = 0.001). The odds of kneeling were worse for a mobile prosthesis compared with a fixed platform design (OR 0.3, 95% CI 0.1–0.7, *p* = 0.005).

**Conclusion:**

A large majority of patients are unable to kneel following TKA, although the ability to kneel improves over time. This evidence may facilitate preoperative patient counseling. Variations in choice of incision location and length may affect ability to kneel; however, high-quality randomized trials are needed to corroborate our findings.

## Introduction

Total knee arthroplasty (TKA) is the definitive surgical treatment for patients with pain and disability attributable to end-stage degenerative knee joint disease. Although a generally successful procedure, a large minority of patients (ranging from 8% to 25%) remain dissatisfied after surgery [1]. While a few of these patients may have suffered from complications—such as infection, instability, or prosthesis loosening—most have well-functioning knees but suffer from so-called nuisance symptoms, among which inability to kneel figures prominently [2].

Kneeling involves placing both knees on the ground and is important for many daily activities. It also holds significant cultural, religious, and occupational value for patients [3–5]. Ninety-four percent of patients expect to be able to kneel 1 year after TKA [6]. Thus, kneeling is of significant importance to TKA candidates and a key element of patient satisfaction. There are few studies that focus on kneeling ability as a primary outcome. Where kneeling has been evaluated, the percentage of patients able to comfortably kneel after TKA has varied greatly from 12% to 90% [7, 8]. Patients who are unable to kneel have cited pain and discomfort, a lack of education on appropriate kneeling technique, and fear of harming their implant as reasons for refraining from kneeling [7]. Further, little research to date has focused on surgical predictors of kneeling ability. To improve outcomes following TKA, it is important to identify the prevalence of kneeling issues among TKA patients and perioperative interventions to improve post-TKA kneeling outcomes.

The purpose of this systematic review and meta-analysis was: (1) to determine the prevalence of kneeling difficulties in patients who have undergone TKA; (2) to identify approaches that may improve kneeling ability; and (3) to quantify the effectiveness of these approaches.

## Methods

This study was conducted according to the guidelines presented in the Preferred Reporting Items for Systematic Reviews and Meta-Analyses statement (PRISMA) [9].

### Assessment of kneeling ability

For studies reporting dichotomous data, kneeling ability was assessed by the number of patients able to successfully kneel. In studies where categorical data on kneeling ability were provided, such as those that used the Knee Injury and Osteoarthritis Outcome Score, or other TKA outcome scales, patients were considered able to kneel if they could do so with mild to no discomfort.

### Eligibility criteria

Studies were eligible for inclusion if patients had undergone primary TKA and postoperative kneeling outcomes data were reported. Any studies not in English were excluded.

### Search strategy

A comprehensive literature search was conducted in MEDLINE and EMBASE from inception to May 2020. Keywords used in the searches were “knee arthroplasty*” OR “knee replacement*” AND “kneel*.” Cited articles were also searched manually to identify any additional studies that were potentially eligible for inclusion.

### Article screening

After executing the search strategy, duplicate articles were removed. The titles and abstracts of the remaining studies were screened by two independent reviewers according to the prespecified eligibility criteria. The remaining studies were then screened using full text by the reviewers. Any disagreements between the reviewers on study inclusion were resolved through discussion and consultation with a second senior author.

### Data extraction

Data were independently extracted by two reviewers into a premade spreadsheet. Information on study characteristics, patient demographics, and kneeling-specific outcomes was noted. Any discrepancies during data extraction were resolved through discussion between reviewers and in consultation with a third reviewer.

### Statistical analysis

Studies were split into two groups based on design. For noncomparative studies, the proportion of patients able to kneel at ≤ 1 year, minimum 1 year, and minimum 3 years follow-up was calculated. Statistical significance based on follow-up times was determined using the *χ*^2^ statistical test. Comparative studies were analyzed separately based on surgical approach or prosthesis design. A random effects model was used to pool outcomes data and determine odds ratios (OR), 95% confidence intervals (CI), and *p*-values. A *p*-value ≤ 0.05 was considered as being statistically significant for all analyses.

### Risk of bias assessment

Study quality was independently assessed by two authors. Randomized control trials (RCTs) were assessed using the revised version of the Cochrane risk-of-bias tool for randomized trials [10]. The remaining studies were assessed for quality using the Methodological Index for Non-Randomized Studies (MINORS) [11].

## Results

### Search results

**Fig. 1:**
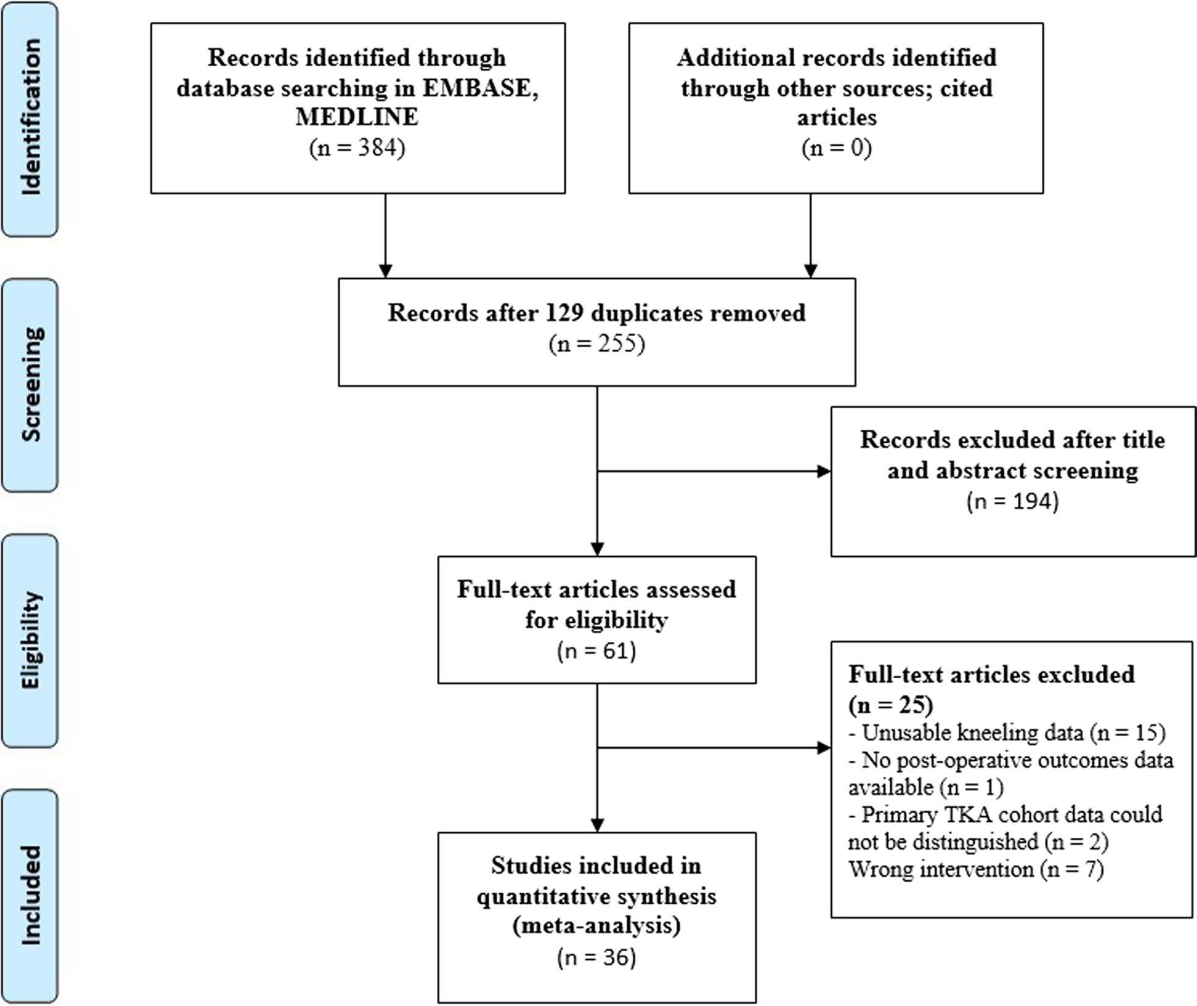
PRISMA flowchart of article selection process

After search retrieval, 384 potential articles were found with 129 duplicates. A total of 255 title and abstracts were screened for eligibility. After the initial screening, 61 studies met the inclusion criteria, and their full text was evaluated. A total of 36 studies were included in the systematic review. A flow chart detailing reasons for study exclusion is provided as Fig. 1.

Approximately 94% of the studies occurred at a single-center institution, with the majority being conducted in Europe (42%). Studies were published between 1999 and 2020 and in total featured 12,626 TKA patients at the latest follow-up time. Twenty-one of the studies were prospective cohort, eight were cross-sectional, five were retrospective chart reviews, and two were RCTs. The number of comparative studies was less than the number of noncomparative studies, 11 versus 25, respectively. All of the studies were written in the English language. A detailed outline of study characteristics is presented in Table 1.

**Table 1:** Summary of study characteristics

Characteristic	Studies, no. (%)
Location	
East Asia	9 (25)
South Asia	1 (3)
Europe	15 (42)
North America	10 (28)
Other	1 (3)
No. of institutions	
Single center	34 (94)
Multicenter	2 (6)
Sample size (TKA patients only)	
< 100	13 (36)
100–400	19 (53)
> 400	4 (11)
Type of study	
Randomized control trial	2 (6)
Prospective cohort	21 (58)
Retrospective cohort	5 (14)
Cross-sectional	8 (22)

### Study quality

For noncomparative studies, the mean MINORS score was 11.2 (range 6–14). The mean MINORS score for comparative studies was 18.4 (range 16–20). In both RCTs, there was some concerns in the selection of reported results, but low risk of bias in all other domains. The overall rating for both RCTs suggested some risk of bias.

### Overall kneeling ability

Thirteen noncomparative studies evaluated kneeling ability at ≤ 1 year follow-up, 24 studies at minimum 1 year follow-up, and 9 studies at minimum 3 years follow-up. The proportion of patients able to kneel increased based on follow-up duration; 34.5% at ≤ 1 year (95% CI 33.5–35.5%), 36.8% at minimum 1 year (95% CI 35.9–37.7%), and 47.6% at minimum 3 years (95% CI 45.6–49.6%). The difference in kneeling ability at 1–3 years and > 3 years follow-up was statistically significant (*p* < 0.001). Overall, the number of patients analyzed at a follow-up time of at least 1 year was 11,514 and 2441 at minimum 3 years follow-up.

### Surgical predictors of kneeling ability

Three surgical approaches showed a statistically significant improvement in kneeling ability after TKA. Kneeling odds were greater for patients undergoing a transverse incision compared with a longitudinal incision (OR 3.5, 95% CI 1.4–8.7, *p* = 0.008), an anterolateral incision compared with an anteromedial incision (OR 3.0, 95% CI 1.3–6.9, *p* = 0.02), and a shorter incision (mean 10.5 cm) compared with a longer incision (mean 18.5 cm) (OR 8.5, 95% CI 2.3–30.9, *p* = 0.001). Only one prosthesis design showed a significant difference in kneeling ability. The odds of kneeling were lower using a mobile prosthesis versus a fixed platform design at 2 years follow-up (OR 0.3, 95% CI 0.1–0.7, *p* = 0.005). A summary of kneeling outcomes for comparative studies is outlined in Table 2 [12–22].

**Table 2:** Pooled kneeling results from comparative studies

Comparison	Differences in ability to kneel
High-flexion versus conventional TKA design	No difference between groups (46% in high flexion TKA versus 44% in conventional TKA, OR 1.1, 95% CI 0.5–2.4, *p* = 0.84) Seon et al. [12]
Patellar resurfacing versus non-resurfacing	No difference between groups (42.7% without resurfacing versus 35.0% with resurfacing; OR 1.6, 95% CI 0.6–4.4, *p* = 0.35) Huish et al. [13]; Garneti et al. [14]
Anterolateral versus midline/medial skin incision	No difference between groups (80.8% with anterolateral incision versus 58.3% with anteromedial incision; OR 3.0, 95% CI 1.3–6.9, *p* = 0.02) Tsukada et al. [15]
Mini-length (mean 10.5 cm) versus standard length (mean 18.5 cm) midline skin incision	Significant difference between groups (40% with MIS versus 0% with standard surgery at 6 months; OR 34.6, 95% CI 1.9–631.9, *p* = 0.02 and 80% with MIS versus 32% with standard surgery at 2 years; OR 8.5 95% CI 2.3–30.9, *p* = 0.001) Kashyap et al. [16]
Transverse versus longitudinal skin incision	Significant difference between groups (70.4% with transverse incision versus 40.6% with longitudinal incision; OR 3.5, 95% CI 1.4–8.7, *p* = 0.008) Ojima et al. [17]
Mobile versus fixed platform design	No difference between groups at 1-year follow-up (25.6% with mobile prosthesis versus 36.4% with fixed design; OR 0.6, 95% CI 0.3–1.2, *p* = 0.14) Kim et al. [18]; Artz et al. [19] Mobile platform inferior to fixed platform at 2-year follow-up (10.8% able to kneel with little or no difficulty with mobile prosthesis versus 27.5% with fixed design; OR 0.3, 95% CI 0.1–0.7, *p* = 0.005) Artz et al. [19]
Two different mobile-bearing prosthesis designs	No difference between groups (37.5% with rotation platform versus 21.1% with mobile design; OR 2.3, 95% CI 0.5–10.1, *p* = 0.29) Nam et al. [20]
High-flexion versus mobile platform design	No difference between groups (40% in high-flexion group versus 36% with fixed design; OR 1.2, 95% CI 0.5–2.7, *p* = 0.68) Seon et al. [21]
Cruciate-retaining versus posterior-stabilized design	No difference between groups (40.0% for cruciate retaining versus 37.5% for posterior substituting design; OR 1.1, 95% CI 0.4–3.1, *p* = 0.84) Zhang et al. [22]

## Discussion

This systematic review aimed to quantify the number of patients able to kneel after TKA and determine the effectiveness of surgical approaches and prosthesis designs in improving kneeling results. Pooled results showed that kneeling ability increased with a longer follow-up duration, with 36.8% of patients able to kneel at a minimum of 1 year follow-up and 47.6% able to kneel at a minimum of 3 years follow-up. Among comparative studies, a shorter incision length greatly improved the odds of kneeling compared with a longer incision (OR 5.6, 95% CI 2.3–30.9, *p* = 0.001), a transverse incision increased the odds of kneeling versus a longitudinal incision (OR 3.5, *p* = 0.008), and an anterolateral incision was superior to an anteromedial incision (OR 3.0, *p* = 0.02). Overall, variations of prosthesis design showed limited changes in kneeling ability, with only a single study demonstrating that a fixed platform design increased kneeling odds compared with a mobile design (OR 3.3, *p* = 0.005).

Prior to TKA, 80–95% of patients have high expectations of being able to kneel after surgery [23]. When high preoperative expectations are not met, they can negatively impact patient satisfaction [24–26]. This study found that approximately two-thirds of patients are unable to kneel after at least 1 year post surgery and around half of patients cannot kneel 3 or more years after TKA. The improvement in kneeling ability with longer follow-up times may be attributable to a number of different reasons. Increased numbness—from sensory nerve damage during surgery—is correlated with poorer kneeling ability and is a common symptom reported by patients unable to kneel [27]. Numbness in the knee joint decreases with longer follow-up times, which may allow more patients to kneel [28]. Moreover, some patients choose not to kneel due to pain and discomfort after TKA [7]. Since a reduction in pain and swelling is greatest in the first year after surgery [29–31], more patients in studies with longer follow-up times may be able to kneel. Discomfort after TKA may also be caused by early instability of the joint, and patients with such complications may not be selected for in studies with longer follow-up times [32].

Variations of incision types showed the greatest benefit in kneeling ability after TKA. Minimally invasive surgery (MIS) with a smaller incision length (mean 10.5 cm) significantly increased odds of kneeling compared with a standard incision (mean 18.5 cm). It is important to recognize that, although MIS may improve functional outcomes, there is a steep learning curve associated with the procedure and significant stress on soft tissues during retraction [33]. Moreover, some studies suggest that the functional benefits of MIS TKA may only last several months to a year [34, 35]. This review also found that an anterolateral incision was superior to an anteromedial incision in terms of kneeling ability. Lateral incisions have been found to pose a smaller risk of damage to the infrapatellar branch of the saphenous nerve compared with a midline incision [36]. Since alteration of skin sensation around the incision decreases kneeling ability [37], lateral incisions may improve kneeling outcomes because of a lower risk of nerve damage. A single study found that a transverse incision improved kneeling ability and scar cosmesis compared with a longitudinal incision [17]. Similar to a lateral incision, a transverse incision is associated with a lower risk of sensory disturbance from damage to the infrapatellar branch of the saphenous nerve. However, this technique requires greater subcutaneous dissection and increases operating time [17]. More high-quality studies are needed to fully understand the benefits and drawbacks of various surgical approaches on kneeling ability.

Variations of prosthesis design and patellar resurfacing failed to demonstrate any considerable improvement in kneeling ability. Artz et al. [19] suggested that a fixed platform design increased the odds of kneeling compared with a mobile design at 2 years follow-up. However, when outcomes data were pooled, no significant differences in the odds of kneeling were noted at 1 year follow-up (OR 0.6, 95% CI 0.3–1.2, *p* = 0.14). Some prosthesis designs, such as high flexion, may offer benefits such as increased range of motion [38]; however, this did not translate to improved kneeling ability. Similarly, although patellar resurfacing did not demonstrate any functional benefit in this study, a meta-analysis suggested that resurfacing can substantially reduce the need for reoperation [39]. Orthopedic surgeons should weigh the benefits and drawbacks of each intervention, in consultation with patients, to improve outcomes.

Although this study investigated surgery-related predictors of kneeling ability post-TKA, rehabilitation programs may also have an effect. Patient education programs on proper kneeling technique have previously shown some success [40]. Moreover, unilateral or bilateral TKA may also influence kneeling ability. In this study, patients with partial TKA were excluded. Future research should also compare kneeling outcomes between these subsets of patients.

### Strengths and limitations

This systematic review has a number of strengths that help to validate our results. The methodology used for this study was in accordance with PRISMA guidelines [9], thereby ensuring that our methods were robust and standardized. Moreover, we broadly searched multiple electronic databases and supplemented our results by manually searching cited articles. A large sample size of 11,614 patients were assessed for kneeling ability in noncomparative studies, thus further strengthening our conclusions for overall kneeling ability.

This study pooled outcomes data from several different articles, thus limiting the results owing to the quality and heterogeneity of the original data. Not all studies reported the same types of outcomes data, and those that did may not be suitable for a meta-analysis. Since kneeling ability was measured in all studies, it is suitable to be included in a pooled analysis model. It is also important to acknowledge that many comparative studies were observational in design or reported a small sample size. As such, findings from such studies should be considered preliminary, and further research is required. It is also important to acknowledge that a patient’s actual kneeling ability may deviate up to 32% from their perceived ability [41]. Thus, the findings of this systematic review are limited due to the variation in kneeling assessment between studies, i.e., patient self-reporting and external validation.

The studies included in this systematic review were subject to varying degrees of bias. Many comparative studies received a low MINORS rating due to unblinded assessment of the endpoint. Although blinding assessors to kneeling ability would not have been difficult, few studies reported this information. As such, it is not possible to determine if the assessment of kneeling ability was unduly influenced by external factors. Similarly, many nonrandomized studies received a low MINORS score due to nonprospective calculation of sample size. It is not possible to determine if kneeling ability was over- or underreported in some studies owing to improper sample size. For both RCTs assessed in this study, there was some concern for bias under the selection of reported outcomes domain. Neither of the RCTs listed a prespecified analysis plan. Therefore, it was not possible to ascertain if reporting bias was present.

## Conclusion

Many patients may not meet their expectations of kneeling ability after TKA, as a large majority of patients are unable to kneel. The ability to kneel tends to improve over time, with significantly more patients able to kneel at a minimum of 3-year follow-up in comparison with 1-year follow-up. This evidence may facilitate preoperative patient counseling.

Limited evidence suggests that variations in choice of incision location and length may affect ability to kneel. High-quality randomized trials are needed to evaluate potential perioperative interventions that can improve kneeling in patients after TKA, and to further corroborate our findings.

## Data Availability

Please contact the corresponding author for information about datasets used in this study.

## Abbreviations

TKA: Total knee arthroplasty
PRISMA: Preferred Reporting Items for Systematic Reviews and Meta-Analyses statement
OR: Odds ratio
CI: Confidence interval
MINORS: Methodological Index for Non-Randomized Studies
RCT: Randomized controlled trial
MIS: Minimally invasive surgery
